# Effect of kisspeptin on *in vitro* maturation of sheep oocytes

**DOI:** 10.14202/vetworld.2017.276-280

**Published:** 2017-03-04

**Authors:** Priyanka Byri, Arunakumari Gangineni, K. Ramachandra Reddy, K. B. P. Raghavender

**Affiliations:** 1 Department of Veterinary Gynaecology and Obstetrics, College of Veterinary Science, P.V. Narasimha Rao, Telangana Veterinary University, Rajendra Nagar, Telangana, India; 2 Department of Veterinary Gynaecology and Obstetrics, College of Veterinary Science, P.V. Narasimha Rao, Telangana Veterinary University, Korutla, Telangana, India; 3 Department of Veterinary Surgery and Radiology, College of Veterinary Science, P.V. Narasimha Rao, Telangana Veterinary University, Rajendra Nagar, Hyderabad, India

**Keywords:** kisspeptin, *in vitro* maturation, sheep oocytes

## Abstract

**Aim:**

The aim of this study was to investigate the effect of kisspeptin (KP) on *in vitro* maturation (IVM) of sheep oocytes aspirated from the ovaries collected from slaughterhouse.

**Materials and Methods:**

Two different experiments were conducted to investigate the effect of KP (5, 10 and 15 µg/ml) alone (experiment 1) or in combination with follicle-stimulating hormone (FSH), luteinizing hormone (LH), and Estradiol (E_2_) (experiment 2) on IVM of sheep oocytes. Tissue culture medium 199 supplemented with Gentamicin was used as control medium. Good quality oocytes were randomly allocated into different IVM media and cultured at 38.5°C in 5% CO_2_ under humidified atmosphere for 24 h. The oocytes were evaluated for their cumulus cell expansion (CCE) and extrusion of the 1^st^ polar body (PB) at the end of maturation.

**Results:**

The proportion of oocytes showing CCE and extrusion of PB was highest when the oocytes were matured in the medium supplemented with 10 µg/ml of KP. In experiment 2, oocytes were matured in 12 different maturation media (G_1_-G_12_: G_1_: Control, G_2_: KP alone, G_3_: FSH, G_4_: FSH+KP, G_5_: LH, G_6_: LH+KP, G_7_: E_2_, G_8_: E_2_+KP, G_9_: FSH+LH+E_2_, G_10_: FSH+LH+E_2_+KP, G_11_: FSH+LH+E2+fetal bovine serum (FBS), G_12_: FSH+LH+E_2_+FBS+KP). The proportion of oocytes showing cumulus expansion and PB extrusion was highest (98.33±1.05 and 89.17±2.38) when they were matured in FSH+LH+E_2_+FBS+KP (G_12_) and was significantly higher than other groups. The proportion of CCE and extrusion of PB was significantly increased when KP was supplemented to FSH and E_2_, but no effect was observed with LH. The maturation rates were significantly increased when FSH, LH, and E_2_ (G_9_) containing media were additionally supplemented with KP (G_10_).

**Conclusion:**

This study demonstrated that the addition of KP (10 µg/ml) to the FSH, LH, and E_2_ supplemented media would enhance the sheep oocyte maturation *in vitro*.

## Introduction

Gonadotropin-releasing hormone (GnRH) has been known to play a major role in the control of reproduction for the last 30 years, but the mechanisms involved in the secretion of GnRH at cellular and molecular level are not completely understood [1]. Recent developments in reproduction with the use of kisspeptin (KP) have opened a new vista to understand these regulatory mechanisms of GnRH secretion [2]. KP seems to be involved in the onset of puberty, initiation of the breeding season and the dynamic changes of follicle-stimulating hormone (FSH) and luteinizing hormone (LH) secretion during estrous cycle [3]. KP, first identified as a metastasis suppressor molecule, is the product of the gene Kiss-1, which encodes a 145-amino acid peptide. It has biologically active peptides ranging from 10 to 54 amino acids in length [4]. Genes encoding KP (KISS1) and its receptors (KISS1R) have been documented in the ovaries of fish [5], hamsters [6], pigs and goats [7], primate, and human ovaries [8].

Administration of KP induces the secretion of gonadotropin hormones in many species such as pigs [9], bovines [10,11], ewes [12-14], goats [15], canines [16,17], and women [18,19]. The ability of KP to induce ovulation following intravenous administration has been demonstrated in prepubertal ewe lambs [20] and anestrous ewes [21]. The presence of high concentrations of KP in the porcine follicular fluid than in serum (335 vs. 25 pg/mL) and expression of KISS1Rs in oocytes and cumulus cells during *in vitro* maturation (IVM) pig oocytes supported the local involvement of KP on the development of oocytes within the follicle [22]. Apart from these *in vivo* studies, very few studies have also been conducted to see the effect of KP on IVM of oocytes. Supplementation of KP to maturation media has been reported to improve the efficiency of pig [22] and bovine [23] oocyte maturation *in vitro*, but there are no studies on IVM of sheep oocytes.

Therefore, taking into account the significance of KP in development of follicles, this study was undertaken to study the effect of KP on IVM of sheep oocytes.

## Materials and Methods

### Ethical approval

The experiments comply with guidelines laid down by the Institutional Ethical Committee.

### Chemicals and media

All media, hormones, fetal bovine serum (FBS), and other chemicals were sourced from Sigma Chemical Co., USA, and plastic ware was from Nunc, Denmark, unless otherwise indicated. KP-10 (Tyr-Asn-Trp-Asn-Ser-Phe-Gly-Leu-Arg-Phe-NH_2_) was purchased from Auropeptides, Hyderabad, India. HEPES buffered tissue culture medium 199 supplemented with 10% FBS (Handling medium) was used for washing and handling of oocytes. Heparin (25 IU/ml) was additionally added to the handling medium for collection of oocytes. Bicarbonate buffered tissue culture medium 199 (TCM199B) supplemented with gentamicin (50 µg/ml) was used as control medium for maturation of oocytes. The media used for transport and washing of ovaries, collection, handling, and maturation of oocytes were supplemented with gentamicin (50 µg/ml) and filter sterilized (0.22 µm) before use. Handling, collection, and maturation media were equilibrated with 5% carbon dioxide in air, in a humidified atmosphere at 38.5°C for at least 2 h before use.

### Collection and IVM of oocytes

Collection and processing of ovaries for aspiration of cumulus oocyte complexes (COCs) were carried out as described by Arunakumari *et al*. [24,25]. Briefly, sheep ovaries were collected from a local slaughterhouse and transported to the laboratory in Dulbecco’s phosphate buffered saline (D-PBS, Gibco, BRL, USA) maintained at 35°C, within 2 h after collection. Each ovary was trimmed off adjacent fat and ligaments. External surface of the each ovary was sterilized by rinsing once in 70% alcohol and thrice in D-PBS. COCs were aspirated aseptically from the visible follicles (>6 mm diameter) using a 20 G needle attached to 5 ml disposable syringe containing 2 ml of collection medium. COCs with homogeneous cytoplasm surrounded by at least three layers of compact cumulus cells were selected for IVM. The selected COCs were washed extensively in handling medium followed by respective IVM media. Subsequently, the 8-10 COCs were transferred into 50 µl droplets of different IVM media in 35 mm culture dishes. The droplets were overlaid with autoclaved pre-equilibrated mineral oil. The dishes with COCs were cultured in an incubator with 5% CO_2_ under humidified air at 38.5°C for 24 h.

### Evaluation of oocytes following IVM

At the end of IVM, the COCs were examined for cumulus cell expansion (CCE). The cumulus cells were removed by incubating them with 100 IU/ml of hyaluronidase for 15 min at 37°C and repeatedly passing them through a fire polished narrow bore glass capillary. The denuded oocytes were examined under an inverted microscope (TH4-200, Olympus, Japan) for the presence of the 1^st^ polar body (PB) in the perivitelline space.

### Experimental design

Two separate experiments were designed. In the first experiment, influence of KP on IVM of the sheep COCs was investigated by supplementing the control medium with 5, 10 and 15 µg/ml of KP. After determining the best concentration of KP, the effect of KP with different hormones was investigated in the second experiment (Table-1).

**Table-1:** Components of IVM media used in the experiment 2.

S. No.	IVM media groups	Composition of different IVM media
1	G_1_	Control medium: TCM 199B+Gentamicin (50 µg/ml)
2	G_2_	Control medium+KP: 10 µg/ml
3	G_3_	Control medium+FSH: 10 µg/ml
4	G_4_	Control medium+FSH (10 µg/ml)+KP (10 µg/ml)
5	G_5_	Control medium+LH: 10 µg/ml
6	G_6_	Control medium+LH (10 µg/ml)+KP (10 µg/ml)
7	G_7_	Control medium+E2: 1 µg/ml
8	G_8_	Control medium+E2 (1 µg/ml)+KP (10 µg/ml)
9	G_9_	Control medium+FSH (10 µg/ml)+LH (10 µg/ml)+E_2_ (1 µg/ml)
10	G_10_	Control medium+FSH (10 µg/ml)+LH (10 µg/ml)+E_2_ (1 µg/ml)+KP (10 µg/ml)
11	G_11_	Control medium+FSH (10 µg/ml)+LH (10 µg/ml)+E_2_ (1 µg/ml)+FBS (10%)
12	G_12_	Control medium+FSH (10 µg/ml)+LH (10 µg/ml)+E_2_ (1 µg/ml)+FBS (10%)+KP (10 µg/ml)

IVM=*In vitro* maturation, KP=Kisspeptin, FSH=Follicle stimulating hormone, LH=Luteinizing hormone, E_2_=Estradiol, FBS=Fetal bovine serum

### Statistical analysis

The proportion of oocytes exhibiting CCE and extrusion of PB were analyzed by ANOVA followed by Duncan’s multiple range test using SPSS software version 16.0 (SPSS Inc., USA) and presented as the mean±standard error.

## Results

In experiment 1, the proportion of COCs showing CCE in all three KP supplemented groups was similar to control, but the proportion of oocytes that extruded PB was significantly higher in KP supplemented media than in control medium (Table-2). Among the KP supplemented groups, the proportion of oocytes that extruded PB was significantly higher when COCs were cultured in the medium supplemented with 10 µg/ml of KP.

**Table-2:** Effect of different concentrations of KP on *in vitro* maturation of sheep oocytes.

Groups	Concentration of KP	Number of oocytes (replicates)	Proportion of CCE	Proportion of extrusion of 1^st^ PB
T_1_	Control (without KP)	120 (10)	68.33±2.42^a^	4.99±1.36^a^
T_2_	05 µg/ml	120 (10)	73.33±1.67^a^	11.67±1.84^b^
T_3_	10 µg/ml	120 (10)	74.17±2.31^a^	24.17±1.45^c^
T_4_	15 µg/ml	120 (10)	71.67±1.84^a^	18.33±1.11^d^

Means with different superscripts, within a column, are significantly different. One-way ANOVA followed by Duncan’s multiple range test (p<0.05). KP=Kisspeptin, CCE=Cumulus cell expansion, PB=Polar body

KP did not show any additional effect on CCE when KP was added to FSH, LH, and E_2_ supplemented (G_4_, G_6_, and G_10_, respectively) and FSH+LH+E_2_+FBS (G_12_) media (Table-3). However, the proportion of COCs exhibiting CCE was significantly increased when KP was added to E_2_ supplemented medium. The number of oocytes that extruded PB was significantly increased when KP was added to FSH, E_2_, FSH+LH+E_2,_ and FSH+LH+E_2_+FBS supplemented media (G_4_, G_8_, G_10_, and G_12_, respectively). Interestingly, KP did not enhance the PB extrusion, in combination with LH (G_6_).

**Table-3:** Effect of KP in combination with FSH, LH, and E_2_ on *in vitro* maturation of sheep oocytes.

Groups	Combinations	Number of oocytes (replicates)	Proportion of CCE	Proportion of extrusion of 1^st^ PB
G_1_	Control	120 (6)	69.17±2.71^b^	6.67±1.05^a^
G_2_	KP-10	120 (6)	76.67±2.47^c^	26.67±2.11^b^
G_3_	FSH	120 (6)	91.67±1.67^e^	54.17±2.71^c^
G_4_	FSH+KP	120 (6)	94.17±0.83^ef^	69.17±3.27^e^
G_5_	LH	120 (6)	84.17±1.54^d^	58.33±4.01^cd^
G_6_	LH+KP	120 (6)	89.17±3.00^de^	64.17±3.01^de^
G_7_	E_2_	120 (6)	52.50±2.14^a^	31.67±2.11^b^
G_8_	E_2_+KP	120 (6)	67.50±1.71^b^	59.17±2.39^cd^
G_9_	FSH+LH+E_2_	120 (6)	90.83±2.01^e^	60.01±2.58^cd^
G_10_	FSH+LH+E_2_+KP	120 (6)	95.01±0.01^ef^	70.01±1.29^e^
G_11_	FSH+LH+E_2_+FBS	120 (6)	95.01±0.01^ef^	80.00±1.29^f^
G_12_	FSH+LH+E_2_+FBS+KP	120 (6)	98.33±1.05^f^	89.17±2.38^g^

Means with different superscripts, within a column, are significantly different. One-way ANOVA followed by Duncan’s multiple range test (p<0.05). FSH=Follicle stimulating hormone, LH=Luteinizing hormone, E_2_=Estradiol, KP=Kisspeptin, CCE=Cumulus cell expansion, PB=Polar body, FBS=Fetal bovine serum

## Discussion

This study has demonstrated for the first time that addition of KP to FSH, LH, E_2_ supplemented media can significantly increase the maturation rates of sheep oocytes *in vitro*. In experiment 1, TCM-199B was supplemented with 0, 5, 10, 15 µg/ml of KP, to identify the best concentration to support oocyte maturation in terms of CCE and extrusion of the 1^st^ PB. In sheep, 8-20 µg of KP was infused to monitor FSH and LH hormones in earlier studies [20,21]. Based on the CCE and extrusion of PB, 10 µg/ml of KP supported better for IVM of sheep oocytes. Similar to the present findings, maturation rate was improved when the bovine oocytes were matured in TCM-199 supplemented with 10 nM of KP (13 µg) [23]. In contrast, none of the pig oocytes extruded PB when maturation medium was supplemented with KP alone [22]. It is likely that the action of KP on cumulus cells and oocytes might be different from species to species. For example, in ovariectomized sheep, the increased LH response to KP administration was decreased by administration of KP antagonist (p234) [26], whereas in dogs it was not altered by KP antagonist (p271) [17]. In this study, the pattern of oocyte maturation significantly increased from 5 to 10 µg/ml and then decreased at 15 µg/ml. *In vivo* secretion of LH in response to KP administration was found to vary with dose, breed and physiological status of animal [13-15,27].

The action of KP on maturation of oocytes *in vitro* is different to that from *in vivo*. *In vivo*, KP directly acts on hypothalamic neurons and results in release of GnRH, followed by FSH and LH, which are responsible for follicle growth and ovulation. The expression of GnRHα and LH on cumulus cells and oocytes during IVM of pig oocytes suggested that KP activates its subordinate GnRHα, thereby increasing LH synthesis, which in turn will affect oocyte maturation [22]. In this study, to see the effect of KP with FSH, LH, and E_2_ alone or in combination, the COCs were matured in different maturation media (experiment 2, Table-1). The addition of FSH, LH, and E_2_ to IVM medium has been shown to improve the maturation rates in sheep oocytes, but the role of each hormone is not clear [28-30]. The proportion of oocytes matured in medium supplemented with KP alone (G_2_) was significantly lower than that of FSH, LH, and E_2_ supplemented groups (G_3_, G_5_, G_7_, and G_9_). In contrast, the bovine oocyte maturation rates in KP supplemented media was similar to that supplementing with FSH and FBS [23]. In this study, the addition of KP to FSH (G_4_) and E_2_ (G_8_) supplemented media significantly enhanced extrusion of PB, compared to the media containing FSH or E_2_ alone, whereas no improvement was observed in LH and KP combination (G_6_) (Table-3). It has been reported that priming of FSH is essential for the action of KP on IVM of pig oocytes. KP alone did not support extrusion of PB (0%), but in combination with FSH (76%) increased extrusion of PB [22]. Similarly, the addition of KP to the E_2_ supplemented medium appears to suppress the negative effect of E_2_ on nuclear maturation as shown in the pig [31].

The proportion of oocytes that showed CCE (95%) and extrusion of PB (80%) was significantly higher in standard FSH+LH+E_2_+FBS supplemented medium (G_11_) than control and other combinations (G_2_-G_10_). This might be due to the presence of FBS in the maturation medium which provides nutrients, vitamins, and growth factors to the cells surrounding the oocyte and therefore plays an important role in the maturation of oocytes [32]. The maturation rates in IVM medium were on par with the maturation of earlier studies in sheep [25,33,34]. However, the addition of KP to the FSH+LH+E_2_+FBS supplemented medium (G_12_) significantly increased the CCE (98%) as well as PB extrusion (89%) when compared to FSH+LH+E_2_+FBS (G_11_). The expression of KP receptors (KISS1Rs) in both oocytes and cumulus cells during IVM of pig oocytes and the increased maturation rates in FSH+KP supplemented media has been shown to be the result of the direct action of KP on oocytes and cumulus cells in an autocrine-paracrine fashion as in folliculogenesis [22]. Addition of various growth factors such as fibroblast growth factor-2, epidermal growth factor, insulin–like growth factor, insulin transferrin selenium, and growth hormone to IVM medium was also found to result in higher maturation and fertilization rates and improved embryo development in sheep oocytes [30,35-37]. Further, the combinations of these growth factors and hormones successfully supported *in vitro* culture of sheep PFs and yielded fertilizable oocytes [24,38] and developed to morulae after IVM and fertilization [25], indicating the synergistic and direct action of hormones and growth factors on *in vitro* culture of sheep PFs and oocytes. The results of this study demonstrated the local interaction of KP with FSH, and E_2_ during IVM of sheep oocytes as hypothesized in earlier studies [22] but it needs to be further explored by molecular approaches. The development competence of the oocytes matured in the presence of KP needs to be further evaluated by *in vitro* fertilization studies.

## Conclusion

This study demonstrated that the addition of KP (10 µg/ml) to the FSH, E_2_, FSH+LH+E_2_ supplemented media would enhance the sheep oocyte maturation *in vitro*. Addition of KP to FSH+LH+E_2_+FBS medium further increased the maturation rates *in vitro*.

## Authors’ Contributions

PB and AG: Conceived and designed the experiments, Performed the experiments. PB, AG, KRR and KBPR: Analysed the data, drafted and revised the manuscript. All authros have read and approved the final manuscript.
